# Combining Evidence, Specificity, and Proximity towards the Normalization of Gene Ontology Terms in Text

**DOI:** 10.1155/2008/342746

**Published:** 2008-03-03

**Authors:** S Gaudan, A Jimeno Yepes, V Lee, D Rebholz-Schuhmann

**Affiliations:** 1 European Bioinformatics Institute, Cambridge CB10 1SD, UK

## Abstract

Structured information provided by manual annotation of proteins with Gene Ontology concepts represents a high-quality reliable data source for the research community. However, a limited scope of proteins is annotated due to the amount of human resources required to fully annotate each individual gene product from the literature. We introduce a novel method for automatic identification of GO terms in natural language text. The method takes into consideration several features: (1) the evidence for a GO term given by the words occurring in text, (2) the proximity between the words, and (3) the specificity of the GO terms based on their information content. The method has been evaluated on the BioCreAtIvE corpus and has been compared to current state of the art methods. The precision reached 0.34 at a recall of 0.34 for the identified terms at rank 1. In our analysis, we observe that the identification of GO terms in the _cellular component_ subbranch of GO is more accurate than for terms from the other two subbranches. This observation is explained by the average number of words forming the terminology over the different subbranches.

## 1. Introduction

Gene Ontology (GO) is a structured database of biological knowledge that provides a controlled vocabulary to describe concepts in the domains of molecular and cellular biology [1]. Currently, the GO vocabulary consists of more than 23 000 terms and is distributed via the GO web pages at http://www.geneontology.org/. GO contains three ontologies: (1) molecular function that describes molecular activities, (2) biological process that describes ordered assemblies of events or a recognized series of molecular functions, and (3) cellular component that describes subcellular locations and macromolecular complexes. Gene products across all species are annotated with GO terms (called GO annotations) to support the description of protein molecular characteristics. These annotations support exchange and reuse of gene characteristics in a standardized way.

The GO annotation (GOA) data is generated by the GO Consortium members through a combination of electronic and manual techniques based on the literature. The manual annotations ensure high-quality reliable data set and have been frequently used by the biological and bioinformatics communities [2]. Apart from manual annotation of proteins, the GO knowledge resource is used to annotate microarray data [3] and to automatically annotate proteins with GO terms amongst other semantic types [4].

However, generation of manual GO annotations based on the literature requires a team of trained biologists and is time consuming, which leads to a limited and thus insufficient coverage of proteins with manual annotations. As a consequence, more efficient means are required to increase the throughput of annotations. Text mining technology is a means to support the annotation process by efficiently identifying and extracting information from the literature and transforming it into GOA input. This approach can produce a significant increase in the number of GO annotations from the scientific publications and improve the benefit from reusing the data.

Automatic GO annotation methods using text mining have been evaluated in the BioCreAtIvE competition as part of task 2 [5]. In the first subtask of task 2, the competition participants had to identify a passage in a full text document that supports the annotation of a given protein with a given GO term.

Different techniques have been applied to this task referenced in Blaschke et al. [5] that can be split into three groups. (1) The first group of techniques processed the GO lexicon to feed the content into pattern matching techniques. (2) The second group used the input information to train machine learning techniques for the identification of relevant passages. (3) Finally, the last group applied techniques that combine pattern matching and machine learning approaches (hybrid methods).

The pattern matching methods (group 1) delivered the highest number of correct annotations but produced many false positives. The hybrid methods returned a lower number of false positives but had the disadvantage of a lower number of true positives. Finally, the machine learning techniques showed intermediate performance. The outcome of the BioCreAtIvE contest is that automatic GO annotation remains a challenging task and that further research is required to obtain better results.

The work presented in this paper is best categorized into the pattern matching group and our results will be compared to two state-of-the-art methods from the same group: Ruch [6] and Couto et al. [7].

Both methods base their scoring functions on the word frequencies extracted from the GO lexicon. The more frequent a word is, the smaller the contribution of the word to the final score of the GO term. Furthermore, Ruch's method increments the score of a GO term for which the candidate matches one of the predefined syntactical patterns of the system while Couto's method limits the look-up of words to the sentences and filters out GO terms that are frequent in the Gene Ontology annotations.

Apart from the contributions to the BioCreAtIvE workshop, other solutions for the extraction of Gene Ontology terms from text have been proposed. GoPubMed [8] is a web-based tool that offers access to subgraphs from Gene Ontology that matches PubMed abstracts. The methods behind GoPubMed identifies GO terms in abstracts by matching first the suffix of the terms and then gathering one by one the more specific words of the left part of the terms. In addition, a selection of general terms' suffixes and some words from a stop list is ignored during the word matching process. The authors do not provide explicit measures of the recall.

Our approach is also based on the identification of weighted words that compose terms denoting GO concepts. The novelty of our method resides in the integration of two new aspects in the scoring method: the proximity between words in text and the amount of information carried by each individual words.

The paper is organized as follows. First, we describe the method used to score mentions of GO terms in text. Then we explain the design of the evaluation method and present the obtained results. Finally, we focus on the characteristics of the method's performance. The evaluation focuses on the identification of GO terms in text and not on the identification of a text passage containing the evidence for a given protein being related to a given GO term.

## 2. Methods

The identification of a term in text requires the localization of the evidence for its mention in the text. The occurrence of words constituting the terms is a reliable evidence. However, this evidence might introduce related terms that are hypernyms or hyponyms of each other. The selection of the most specific term avoids loss of detail. Evidences extracted from text are unlikely to have a relationship with each other if they are far from each other in text. As a result, such evidence is unlikely to be the result of the mention of the term they constitute. Finally, the described methods make abstraction of the order of the words occurring in the terms, thus allowing to identify syntactical variants of the terms in the text.

We describe in the following a method that integrates the concepts of *evidence*, *specificity*, and *proximity* to identify mentions of terms in text.

Before describing all three aspects of the method, the concept of a zone needs to be introduced. A zone is a continuous stretch of text that is composed of words. The decomposition of a document into zones can follow various definitions. The zones of a document can be its paragraphs, its sentences, or, for instance, the noun phrases contained in the document. The zone can also be the document itself.

### 2.1. Evidence and Specificity

The following calculations are inspired from the similarity definition introduced in Lin [9]. The similarity between two entities is defined as the ratio between the amount of information shared by the two entities and the amount of information needed to describe the two entities:(1)

Lin [9] illustrates the benefit from the similarity measure with a number of scenarios such as the assessment of string similarity based on -grams or measurement of the similarity between concepts derived from an ontology by considering the ontology's structure. But more generally, the proposed approach can be applied to any model for describing the entities to be compared. In our study, the units describing the entities are words.

We introduce the technique used to measure the evidence that a term  is mentioned in a zone , and we provide at the same time the description of the specificity measurement of a term.

A term consists of words where each word contributes to the syntactic and semantic representations of the term. Intuitively, the word "" is of lower importance than the word "" to convey the meaning of the term "." Similarly, "" contributes less than "."

Deciding whether a term  is mentioned in a zone  consists of recognizing the individual words of  in  that are necessary to preserve reasonably well its original meaning.

Independently of the text, measuring the contribution of each word in the meaning of a term can be estimated by measuring the amount of information carried by each individual word and comparing it to the global amount of information carried by the term. The amount of information carried by a word  is(2)

where  is the probability that  occurs in a corpus. Several types of corpus will be presented in Section 3. The formula illustrates that a frequent word carries a low amount of information whereas a rare word is rich in information. Considering the amount of information carried by words has proved to be of great benefit with the broadly used TFIDF in the information retrieval field:(3)

Under the assumption that the occurrences of the words in a term are independent, the amount of information carried by a term is(4)

where  is the set of unique words, also called tokens, in the term . The amount of information carried by a term is a suitable measurement of the specificity of the term. However, the specificity of a term can also be estimated by considering the probability that the term itself occurs:(5)

Terms frequencies can nevertheless be nil, thus carrying the same undetermined amount of information. These nil frequencies can be observed for half of the Gene Ontology terms. Therefore, we will privilege the first estimation of the specificity.

Finally, the amount of information from a term  present in a zone  is(6)

The proportion of  in  is the proportion of information carried by the words  present in a zone  and constituting the term . This ratio is used to measure the amount of evidence that a term  occurs in a zone :(7)

### 2.2. Proximity

The fact that the words of a term  occur in a text zone  does not necessarily mean that the term is mentioned in the zone, especially if the zone is relatively large (e.g., the whole document). The proximity between the words of  in  provides a clue on the likelihood that those words belong to a common term.

The proximity of the words forming a term and found in the text is a function of the positional index of the words in the text. The benefit of using proximity between keywords has been greatly shown in the field of information retrieval [10]. Various approaches for measuring the notion of proximity or density have been proposed in order to score and rank more efficiently query results. In the following, we propose to use the proximity in the sense of density.

The relative distances between the individual tokens from the term and the minimum distance between the words in the optimal case where the words are consecutive are taken into consideration when measuring the proximity.

Let  be the set of words of term  found in the zone :(8)

where  is the number of words in . The scatter of the words  in the zone  is the sum of the distances between the individual words of :(9)

where  is the distance measured in words, between the word  and the word  in the zone .

The minimum dispersion for  in  takes place when the words of  are consecutive. Following the previous definition, the minimum dispersion can be directly computed as follows:(10)

We define the proximity of the words  in the zone  as the ratio between the minimum dispersion of  and the dispersion of  in :(11)

The proximity indicates whether the words constituting the terms and found in a zone  are close to each other or dispersed over the zone. The proximity is  when the words of  are consecutive in  and decreases towards , while the words move away from each other in .

In case a word occurs several times in a zone, the smallest distance from all combinations is considered. This solution gives the opportunity to keep a relatively high proximity for a term such as "" that occurs in a zone like "."

For such a case, the determining parameter is the distance between "" and "."

### 2.3. Score

Evidence, specificity, and proximity are the three parameters that are combined to score the mention of a term  in a text zone . The three criteria may be of various importance and must be weighted accordingly. Their importance varies in function of the nature of the terminology and of the text.

The three criteria are combined by the product of the functions, thus providing a veto to the functions. For instance, if no word carries evidence that a GO term is mentioned in the zone, then the score will consequently tend to zero. Similarly, if the words supporting the mention of the term are greatly scattered over the text, then the score will tend to zero, whatever evidence found in the zone. Finally, the three aspects are weighted by using the power of the individual function:(12)

Increasing the power of a function (,  or ) increases the role played by the corresponding criterion.

### 2.4. Previous Work

We provide the details of Couto and Ruch's methods. Our approach is compared to these two systems in Section 3.

Couto et al. [7] score the mention of GO terms in text by considering the "evidence content" of the individual words constituting the GO terms:(13)

where  is the number of ontology terms containing the word  and where  is the sum of  for all the words  occurring in the ontology.

The evidence content of a term is defined as the highest evidence content of its names:(14)

where(15)

Then, they computed the "local evidence content" that a name  is mentioned in a zone :(16)

Again, the local evidence content of a term  is defined as the maximum local evidence content of its names:(17)

Finally, the confidence that a term  occurs in a zone  is defined as the ratio between the local evidence content of the term in the zone and the evidence content of the term:(18)

Upstream the computation of the confidence, some words are discarded from the term weighting by applying a stop list on words such as "" or "."

The approach of Ruch [6] is based on pattern matching and text categorization ranking. The pattern matching module measures the similarity between a term and a sliding window of five words from the passages. The text categorization module introduces a vector space that provides a similarity measure between a GO term, seen as a document, and a passage, seen as a query. The modules ranking the GO terms given a passage as a text categorizer would rank documents given a query. A fusion of the two modules is operated by using the result of the vector space as a reference list while the pattern matching result is used as a boosting factor. Finally, the candidates scores are reordered by considering the overlap between the candidates and the noun phrases extracted from the text. The noun phrases are identified by applying a set of predefined patterns on the text's part-of-speech tags.

## 3. Evaluation

We illustrate here the identification of terms derived from the Gene Ontology (GO). GO provides a controlled vocabulary to describe genes and proteins in any organism. Each entry in GO has, among other fields, a term name, a unique identifier, and a set of synonyms.

The evaluation has the goal to provide a measure of the accuracy of the system for identifying in text terms derived from the Gene Onology.

### 3.1. Method

The system has been evaluated on the BioCreAtIvE corpus, the most suitable standard evaluation set for the annotation of text with Gene Ontology terms. The evaluation illustrates the performance in terms of precision and recall.

The BioCreAtIvE corpus, named here , contains  entries, each of them containing, among other fields, a passage, as well as the GO identifier of the term mentioned in the passage's text.

For each passage, the evaluated system provides a list of candidates, sorted by the score . The precision and recall are computed over all the passages by considering the first  suggestions with . The precision and recall, at rank , are computed as follows:(19)

where  is the set of candidates at rank  and  denotes the correct ones. When  increases, the number of candidates also increases whereas the number of entries  is independent of .

Clearly,  and  are relevant indicators of the performance of the system for a given rank . Nevertheless, comparing several systems requires a global approach that takes into account the performance of the systems at various ranks. We, therefore, introduce a novel measure that assesses the performance of the systems over all the ranks by computing the proportion of correct candidates by the number of entries. Each correct candidate is inversely weighted by its rank. The contribution of a correct candidate at rank  is  unit when a correct candidate at rank  contributes of one fifth. The global performance () measures the accuracy of the system, over all the ranks, and is by consequence suitable for comparing systems against each other,(20)

### 3.2. Statistical Significance

The precision and recall are well-established measurements for estimating the accuracy of text mining methods. However, the comparison of individual methods by only considering the precision/recall can often underestimate their differences [11]. Therefore, while presenting the performance of the individual methods, we also provide a statistical significance of the differences between the precisions of the various methods at rank . To do so, we test the null hypothesis:

Method 1 and method 2 are not different.

Then, we estimate the probability that the precision on the test set is as good as the one return by the so-called best method. To estimate this probability, we first generate  trials where the passage annotations are randomly selected from one of the two methods. For each trial, the precision at rank  is computed. Finally, we compute the maximum probability that we observe a precision that is at least as good as the one of the so-called best method (see [11] for further details):(21)

where  is the number of trial with a precision that is at least as good as the one of the so-called best method and where  is the number of trials. It has been acknowledged that a probability below  illustrates a statistical significance in the result differences [11].

### 3.3. Probabilistic Model

Three probabilistic models have been tested for computing . The first one is based on the word frequencies in Medline. The second one is also based on word frequencies in Medline, but only for abstracts that have been annotated by curators in mammalian annotation projects [12]. The last probabilistic model is based on the word frequencies in the Gene Ontology itself, as in Ruch and Couto's methods.

#### 3.3.1. Medline

The first probabilistic model used to compute  is derived from the document frequencies in Medline:(22)

where  is the number of documents in Medline where  occurs and  is the total number of documents in Medline.

#### 3.3.2. Medline Abstracts from Gene Ontology Annotations (GOA)

This model is based on the document frequency from the GOA and contains around 22 000 references to Medline entries,(23)

where  is the number of GOA documents where  occurs and  is the total number of GOA documents.

#### 3.3.3. Gene Ontology

The last model is based on the word frequencies in the Gene Ontology:(24)

where  is the number of names in the Gene Ontology where  occurs and  is the total number of names in the Gene Ontology. The probability that a word occurs in the ontology is independent of the hierarchical structure of the ontology. Indeed, the structure does not contain information on the probability that a word occurs in a concept name. However, the opposite is not true; it can be observed that many GO terms contain words that also belong to their parents, thus the concept names provide some information on the structure of the ontology. Lin [9] uses the structural information contained in the ontology to measure the semantic similarity between two concepts. However, in the present context, this structural information is not available for the zone and thus cannot be considered. Furthermore, the aim of our approach is not to measure the semantic similarity but the similarity in terms of evidence.

The three models have been evaluated with the optimal parameters (, ) described in the next section. The best performance is reached when the Gene Ontology is used as the model, with a global performance of . Then,  when the Gene Ontology Annotation's corpus is used and decreases even more () with the entire Medline as the model.

### 3.4. Evidence, Proximity, and Specificity

To illustrate the role played by each aspect of the method, we explore the evolution of the precision/recall when the evidence, the specificity, and the proximity are taken or not into consideration. We also consider in the evaluation different types of zones. The following setups have been measured.

(1) Default setup: , , each passage is a zone.

(2) Zone = sentence.

(3) Zone = noun phrase.

(4) Evidence: 

(5) Without proximity ().

(6) Without specificity ().

Setups (1), (2), and (3) illustrate the role of the zone while setups (4), (5), and (6) demonstrate the importance of the evidence, the proximity, and the specificity, respectively. For the sake of brevity, only the optimal parameter is provided. The optimal parameters have been determined by trying the different combinations of ,  and  within  with a step of  on the second independent annotated corpus from BioCreAtIvE.

**Figure 1:**
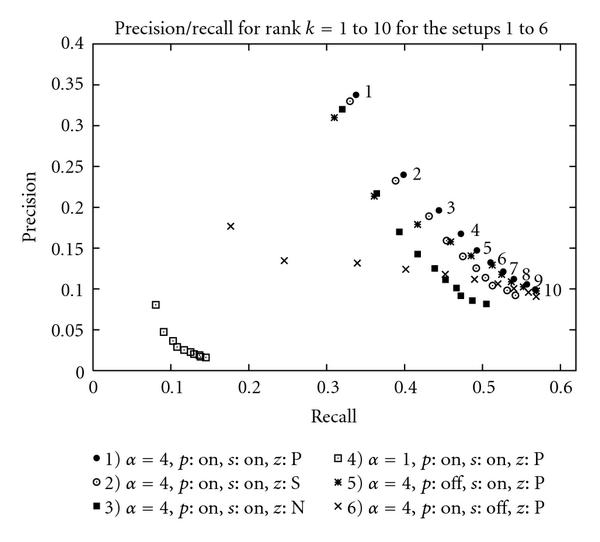
**Precision/recall of the setups for rank  to **. Code:  = proximity,  = specificity, and  = zone.

Figure 1 shows the precision/recall of the system in the setups (1) to (6). The optimal setup is found when  and when the specificity and the proximity are integrated into the final score's equation with the passages as zones. The system achieves a precision/recall of  at rank 1. Table 1 shows the global performance () for each setup.

**Table 1:** Global performance for the six setups.

Setup	
(1) Optimal setup	
(2) Zone = sentence	
(3) Zone = noun phrase	
(4) *α*=1	
(5) Without proximity	
(6) Without specificity	

### 3.5. Comparison with Existing Methods

In order to provide a baseline in the comparison, the exact match method, in other words the identification of terms by the localization of the constituting words in their consecutive order, is evaluated. The method is compared to the exact match method as well as the ones described by Rush [6] and Couto et al. [7].

**Figure 2:**
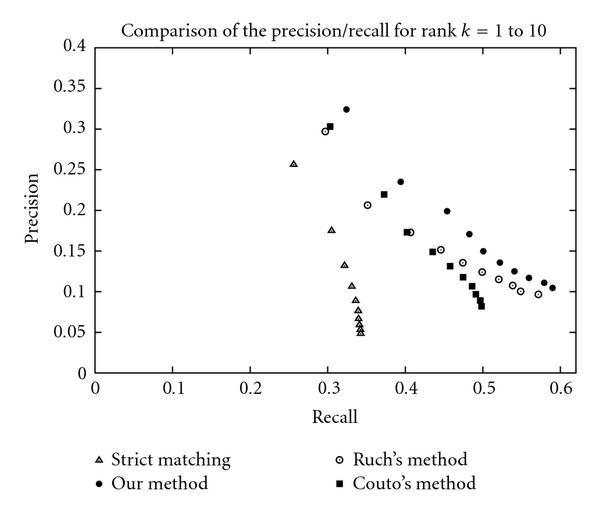
**Comparison of the precision/recall for rank  to **.

The BioCreAtIvE corpus has been generously tagged by the authors of both papers mentioned above and homogeneously evaluated in terms of global performance, precision, and recall. The global performance of the systems illustrated in Figure 2 shows the improvement of our method (precision/recall of  at rank ) over Ruch () and Couto's methods (). However, their methods outperform greatly the strict matching technique () by allowing flexibility on the order of the words as well as their consecutiveness. Table 2 gives the global performance of each method. The differences of performance among the methods that can be observed from the comparison are confirmed by the statistical significance test for rank 1 (Table 3). We can note the convergence of the precision/recall points for higher ranks between our method and Ruch's method (Figure 2). Nevertheless, this phenomenon remains natural since all methods are "word-evidence" based.

**Table 2:** Global performance of the 4 methods.

Method	
Strict matching	
Our method	
Ruch's method	
Couto's method (GO Figo)	

**Table 3:** Probability that the two methods are not different in terms of precision at rank . A probability under  indicates a statistical significance difference between the two methods.

Method	
Ruch's method/Couto's method	
Couto's method/presented method	
Ruch's method/presented method	

### 3.6. Branches of GO

**Figure 3:**
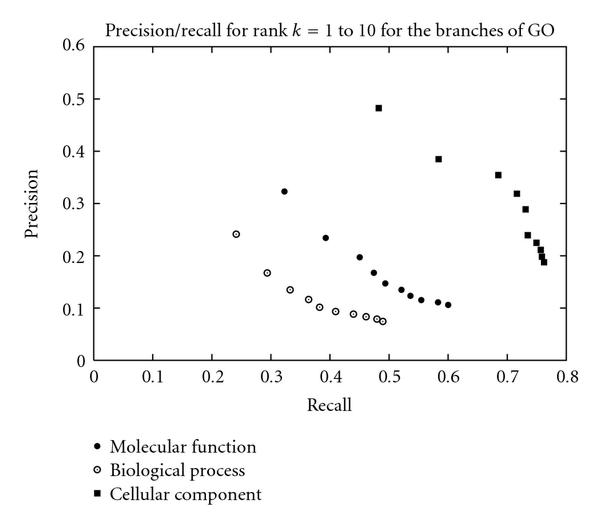
**Precision/recall for the three branches of GO for rank  to **.

The performance of the method is significantly different for each individual branch of the Gene Ontology. Figure 3 and Table 4 illustrate the precision/recall and the global performance for each branch. Terms from the cellular component branch are by far the most often recognized terms with . The molecular function branch is a more difficult terminology to identify in text with . The lowest performance is met on the biological process branch with 

**Table 4:** Global performance for the three branches of GO.

Branch	
Molecular function	
Biological process	
Cellular component	

The same differences are observed on the performance of Rush [6] and Couto et al. [7].

## 4. Discussion

Three probabilistic models have been evaluated and compared. The model based on the Gene Ontology appears as the most suitable. The benefit of using the Gene Ontology as the probabilistic model is explained by the fact that the model is close to the distribution of words from GO in text and provides a good measurement of the information carried by individual words. Medline refers to a heterogeneous set of topics in the biomedical domain. As a consequence, the distribution of words is less suitable. The subset of Medline that is used to annotate proteins with GO terms provides better results because the corpus is more related to the biomolecular domain (Table 5).

**Table 5:** Global performance of the three models.

Probabilistic model	
Gene Ontology	
GOA corpus	
Medline corpus	

The role played by each individual aspect of the method is clearly illustrated in Figure 1. The evidence and the proximity are factors to decide whether the term is mentioned or not in the analyzed text whereas the specificity prioritizes the terms in the list of candidates. As expected, the evidence, in other words the proportion of information carried by words from  found in , plays a major role in the identification of mentioned terms in text. But it also appears that the specificity of the terms is an important criterion when annotating text with GO terms. The proximity is essential when the zone's size increases and is a convenient technique to constrain words to occur in a common window.

The novelty of our method resides in the specificity and the proximity aspects. The integration of these two aspects justifies the better performance over Rush [6] and Couto et al. [7]. In spite of the fact that Couto limits the look-up to sentences and that Ruch increases the score of a pattern match, the notion of a flexible proximity is absent from their methods.

Better performance for the identification of GO annotations has only been reported in two cases that clearly differ from our approach. In Couto et al.'s approach [13], prior knowledge from GOA for selected proteins was used to annotate orthologs based on the identification of GO terms from Medline abstracts with Couto's method. Shatkay et al. [14] transformed Medline abstracts into feature vectors of distinguishing terms that are predictive for a subcellular localization and these feature vectors were used to categorize proteins into a subcellular location. The method appears to be efficient in predicting cellular localizations of proteins when combined with other genomic and proteomic data. However, this method did not propose any evidence of a GO term from the text and is restricted to the cellular localization.

**Figure 4:**
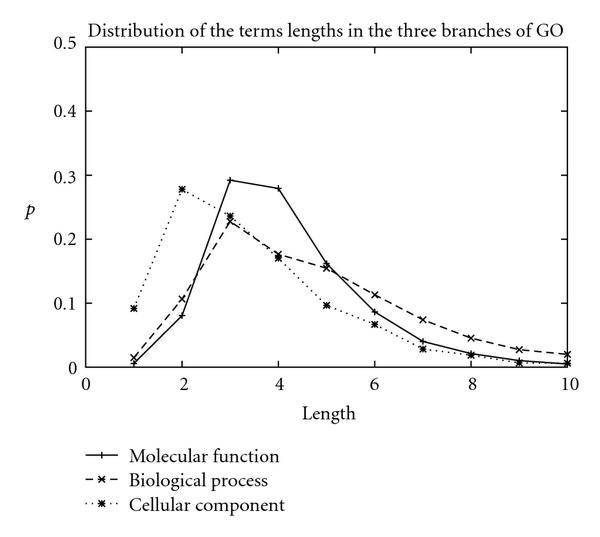
Distributions of the number of words for each branch:

The distribution of the number of words per term in the individual branches of Gene Ontology might play a role in the performance of the systems. Indeed, Figure 4 shows the distributions of the number of words in terms for the individual branches of GO. The biological process branch, which is the most difficult category to identify in text, has on average a higher number of words than the cellular component for which the identification of terms in text is more satisfactory. The chances of identifying all the words of a term can be lower for a long term than a short term. Furthermore, the comprehensive list of synonyms for long terms can be missing from the lexicon given the number of combinations for all the synonyms of the constitutive words of terms (Table 6). Also, terms referenced in the Gene Ontology can represent complex biological concepts that include relationships between various biological entities. The mention of those complex concepts in text can be formulated in an unlimited number of possible manners, thus making the capture of all those mentions almost impossible in an automatic manner. In addition, terms kept in GO have not been collected or designed to fulfill information extraction demands. In particular, GO terms can either refer to some conceptual ideas that are not explicitly mentioned as such in text or, on the contrary, can be often named in the literature under some common forms that are not referenced in GO.

**Table 6:** Examples of GO terms that have not been extracted from the passages.

thiamin-triphosphatase activity:


cytoplasm:

negative regulation of JNK cascade:



sulfate adenylyltransferase (ATP) activity:




Figure 5 clearly shows an uneven distribution of the GO terms occurrences in Medline abstracts by applying our term identification method. It can also be seen that selected entire subbranches consist of frequent terms while terms from some other subbranches are rarely mentioned in Medline abstracts. Interestingly, only  of the GO terms of the molecular function branch are found at least once in Medline, suggesting that the GO terms names are not necessarily optimally defined to match the literature. But surprisingly,  of the GO terms that annotate human proteins are also found in Medline abstracts. This large overlap between the GO terms used in the literature and in the GO annotation database drives the hope to bring both resources close together and to automatically annotate proteins with a sufficient coverage.

**Figure 5:**
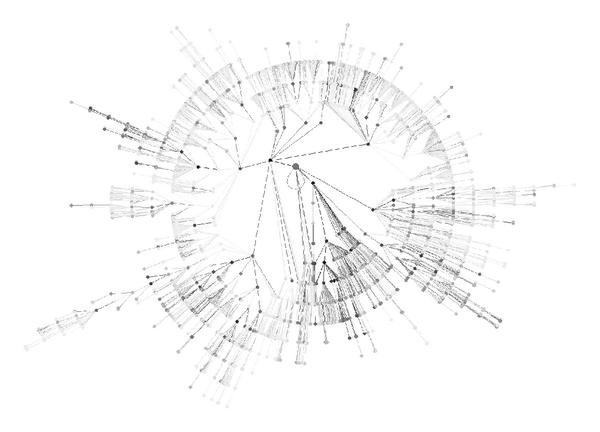
**Graph representing the Gene Ontology structure for the molecular function branch**. The level of gray of a node is function of the frequency of its corresponding GO term in Medline abstracts.

The overall precision of  for a recall at  at rank  seems at first limited. In the context of BioCreAtIvE, the evaluation method judges as correct a GO term only if there exists an annotation between the GO term and the protein mentioned in the passage. However, many other GO terms appear in the passages and are classified as incorrect because they are not bound to a protein. Furthermore, GO terms are refereed with unambiguous names due to the descriptive nature of the names. As a result, if the evaluation focusses only on the mentions of the GO terms, independently of a potential relationship with a mentioned protein, then the recall gets close to 0.6 at rank 10 while the precision can be assumed to get close to 1 due to the unambiguous nature of the terminology. Indeed, the occurrence of a GO name in text has a high probability to refer to the GO concept instead of another concept sharing the same name. In contrast, this property is not observed with protein names due to their ambiguous nature. For instance, the mention of "why" can refer to the adverb or to a gene.

The presented evaluation provides nevertheless an order on the performances of the various methods. It also provides a lower boundary for the precision as well as an upper boundary for the recall on the BioCreAtIvE test set. In other words, the identification of GO terms, independently of a relationship with a protein, can be achieved with a precision close to one and with a recall of 0.6. Such performance provides satisfying results for assisting biologists in the task of curating manuscripts when the localization of Gene Ontology terms in text is needed. The remaining challenging task is then the association of the GO terms with the proteins they potentially describe. This last step is still performed by curators since automatic methods remain deceiving.

## 5. Conclusion

The automatic identification of GO terms using the biomedical literature can significantly increase the amount of information about proteins available as a structured knowledge resource. We composed a new method integrating the notions of evidence, specificity, and proximity to identify terms from text. The evidence is the proportion of statistically meaningful words from a term found in text. This criterion has been previously exploited to achieve the identification of GO terms in text. The specificity allows the selection of the most informative terms while the proximity provides a clue about the belonging of words found in text to a term.

Adding proximity and specificity into the scoring function drives the system to reach better performance in comparison to other state-of-the-art methods. All has been evaluated against the annotated BioCreAtIvE I corpus and has been benchmarked to a precision of 0.34 at a recall of 0.34 for the terms delivered at rank 1. The evidence parameter is the major criterion for scoring mentions of GO terms in text. However, the specificity and the proximity are important to improve the identification of the terms. The size of the zone selected to identify the words has an impact on the performance of the method with an optimal setup based on the passage for the evaluation. The optimal probabilistic model was used to compute the probability that the word occur is based on the Gene Ontology, model also chosen by Couto et al. [7].

Some variations in the performance regarding the individual branches of GO can be observed and might be correlated with the nature and the distribution of the number of words in their respective terms. The performance of the compared systems indicates that further research is needed to better identify terms from the biological process and molecular function branches. Disambiguation, synonym formation, and word variation could potentially improve the performance of the identification of GO terms.
